# Targeting SUMO Modification of the Non-Structural Protein 5 of Zika Virus as a Host-Targeting Antiviral Strategy

**DOI:** 10.3390/ijms20020392

**Published:** 2019-01-17

**Authors:** Zheng Zhu, Hin Chu, Lei Wen, Shuofeng Yuan, Kenn Ka-Heng Chik, Terrence Tsz-Tai Yuen, Cyril Chik-Yan Yip, Dong Wang, Jie Zhou, Feifei Yin, Dong-Yan Jin, Kin-Hang Kok, Kwok-Yung Yuen, Jasper Fuk-Woo Chan

**Affiliations:** 1 State Key Laboratory of Emerging Infectious Diseases, The University of Hong Kong, Pokfulam, Hong Kong Special Administrative Region, China; zhengzhu@hku.hk (Z.Z.) hinchu@hku.hk (H.C.); yuansf@hku.hk (S.Y.); jiezhou@hku.hk (J.Z.); khkok@hku.hk (K.-H.K.); 2 Department of Microbiology, Li Ka Shing Faculty of Medicine, The University of Hong Kong, Pokfulam, Hong Kong Special Administrative Region, China; wenlei07@hku.hk (L.W.); kchik929@connect.hku.hk (K.K.-H.C.); ttyuenii@connect.hku.hk (T.T.-T.Y.); yipcyril@hku.hk (C.C.-Y.Y.); u3004628@connect.hku.hk (D.W.); 3 Hainan Medical University-The University of Hong Kong Joint Laboratory of Tropical Infectious Diseases, Hainan Medical University, Haikou, Hainan, and The University of Hong Kong, Pokfulam, Hong Kong Special Administrative Region, China; yinfeifeiff@163.com; 4 Department of Pathogen Biology, Hainan Medical University, Haikou, Hainan 571101, China; 5 Key Laboratory of Translational Tropical Medicine, Hainan Medical University, Haikou, Hainan 571101, China; 6 School of Biomedical Sciences, The University of Hong Kong, Pokfulam, Hong Kong Special Administrative Region, China; dyjin@hku.hk; 7 Carol Yu Centre for Infection, Li Ka Shing Faculty of Medicine, The University of Hong Kong, Pokfulam, Hong Kong Special Administrative Region, China; 8 The Collaborative Innovation Center for Diagnosis and Treatment of Infectious Diseases, The University of Hong Kong, Pokfulam, Hong Kong Special Administrative Region, China

**Keywords:** antiviral, flavivirus, inhibitor, interferon, NS5, post-translational modification, SUMO, Zika

## Abstract

Post-translational modifications of host or viral proteins are key strategies exploited by viruses to support virus replication and counteract host immune response. SUMOylation is a post-translational modification process mediated by a family of ubiquitin-like proteins called small ubiquitin-like modifier (SUMO) proteins. Multiple sequence alignment of 78 representative flaviviruses showed that most (72/78, 92.3%) have a putative SUMO-interacting motif (SIM) at their non-structural 5 (NS5) protein’s *N*-terminal domain. The putative SIM was highly conserved among 414 pre-epidemic and epidemic Zika virus (ZIKV) strains, with all of them having a putative SIM core amino acid sequence of VIDL (327/414, 79.0%) or VVDL (87/414, 21.0%). Molecular docking predicted that the hydrophobic SIM core residues bind to the β2 strand of the SUMO-1 protein, and the acidic residues flanking the core strengthen the binding through interactions with the basic surface of the SUMO protein. The SUMO inhibitor 2-D08 significantly reduced replication of flaviviruses and protected cells against ZIKV-induced cytopathic effects in vitro. A SIM-mutated ZIKV NS5 failed to efficiently suppress type I interferon signaling. Overall, these findings may suggest SUMO modification of the viral NS5 protein to be an evolutionarily conserved post-translational modification process among flaviviruses to enhance virus replication and suppress host antiviral response.

## 1. Introduction

Zika virus (ZIKV) is a member of the genus *Flavivirus* in the family *Flaviviridae* [1]. It is an enveloped virus with a non-segmented, single-stranded, positive-sense RNA genome which measures around 10 kilobases [1]. Similar to other flaviviruses, the ZIKV polyprotein is cleaved into 10 structural and non-structural proteins, with the coding region orders and non-structural protein motifs being arranged in the order of 5’-Capsid-preMembrane-Envelope-NS1-NS2A-NS2B-NS3-NS4A-NS4B-NS5-3’ [1]. Phylogenetically, it is closely related to other human-pathogenic mosquito-borne flaviviruses, including dengue virus (DENV), yellow fever virus (YFV), West Nile virus (WNV), and Japanese encephalitis virus (JEV) [1]. Initially considered to be geographically confined to parts of Africa and Asia, a large outbreak involving 73% of the population on Yap Island of the French States of Micronesia occurred in 2007 [2]. While most ZIKV-infected patients have mild disease, some may develop severe complications, including congenital microcephaly and malformations in infected fetuses, and Guillain-Barré syndrome, meningoencephalitis, myelitis, thrombocytopenia, disseminated intravascular coagulation with hemorrhagic complications, hepatic dysfunction, orchitis, acute respiratory distress syndrome, shock, and multi-organ dysfunction syndrome in infected adults [3,4]. Because of its clinical importance and rapid spread, the ZIKV epidemic was declared a public health emergency of international concern by the World Health Organization between 1 February 2016 and 18 November 2016 [4,5]. More than 80 countries/territories in the Americas, Africa, and Asia have reported evidence of local vector-borne ZIKV transmission [5].

The strategies utilized by ZIKV to evade the host immune response and replicate efficiently in a broad range of human cell types to cause these protean clinical manifestations are incompletely understood [6,7]. Post-translational modifications of host or viral proteins have been increasingly recognized as key strategies exploited by viruses to support virus replication and counteract the host immune response. SUMO modification of proteins is a post-translational modification process mediated by a family of ubiquitin-like proteins known as small ubiquitin-like modifier (SUMO) proteins [8]. Four isoforms of SUMO proteins, namely, SUMO-1, -2, -3, and -4, are found in mammals. SUMO-1 shares low (50%) sequence identity with SUMO-2 and -3, which are structurally highly identical (97% sequence identity) to each other [8,9,10]. SUMO-1 and SUMO-2/3 have distinct functions, whereas the role of SUMO-4 remains undetermined [8,11]. The binding of these SUMO proteins to their target proteins induce conformational changes that hinder or create binding sites to its interactors [12]. SUMO modification of proteins is involved in the regulation of a wide variety of cellular processes, including protein subcellular localization, transcription, DNA repair, chromosome dynamics, and stabilization of modified proteins [8,13,14,15,16]. Increasing evidence indicates that SUMO modification of viral or host proteins is involved in the regulation of virus-host interactions and can affect the replication of various viruses, including influenza viruses, hepatitis D virus, picornaviruses, rhabdoviruses, and retroviruses, through direct modification of viral proteins or modulation of the host antiviral response [8,17,18].

Recently, a SUMO-interacting motif (SIM) at the N-terminal domain of the non-structural 5 (NS5) protein of DENV was identified and the DENV NS5 protein was validated to be a SUMOylated protein [19]. SUMO modification of the DENV NS5 protein stabilizes the protein to support virus replication and suppresses the innate host immune response [19]. As the NS5 protein is highly conserved among flaviviruses, we therefore hypothesized that SIMs similar to the one found in the DENV NS5 protein may also be present in the NS5 proteins of ZIKV and other flaviviruses [1]. In this study, we investigated for the presence of SIMs at the NS5 protein of ZIKV and other flaviviruses, and evaluated the anti-ZIKV effect of the SUMO inhibitor 2-D08.

## 2. Results

### 2.1. The Putative SIM at the N-Terminal Domain of NS5 Protein Is Highly Conserved among Flaviviruses

**Figure 1:**
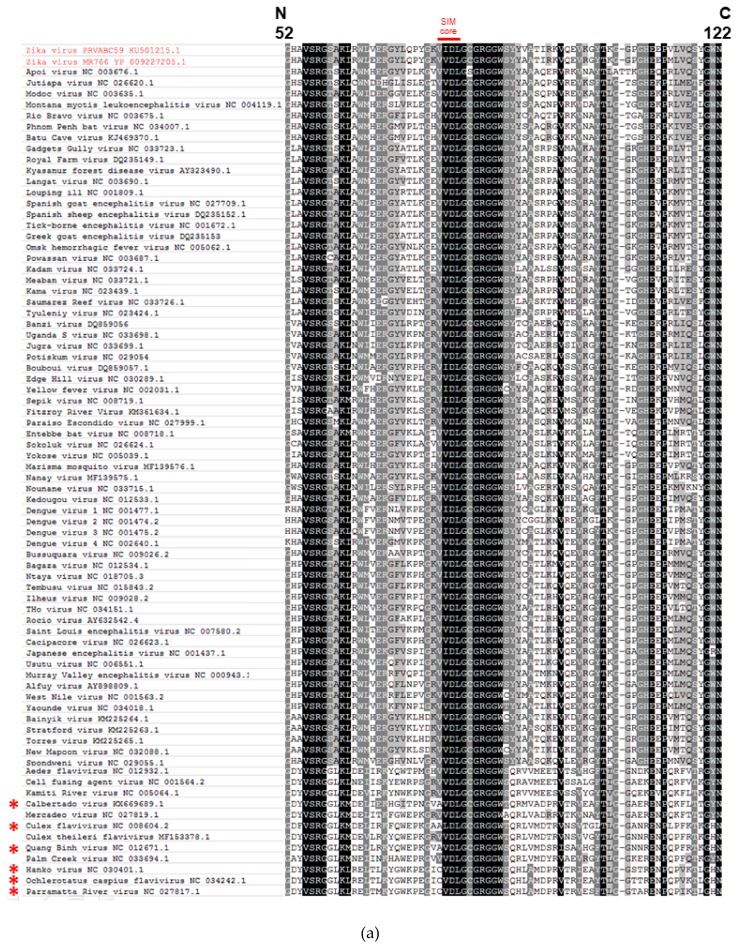

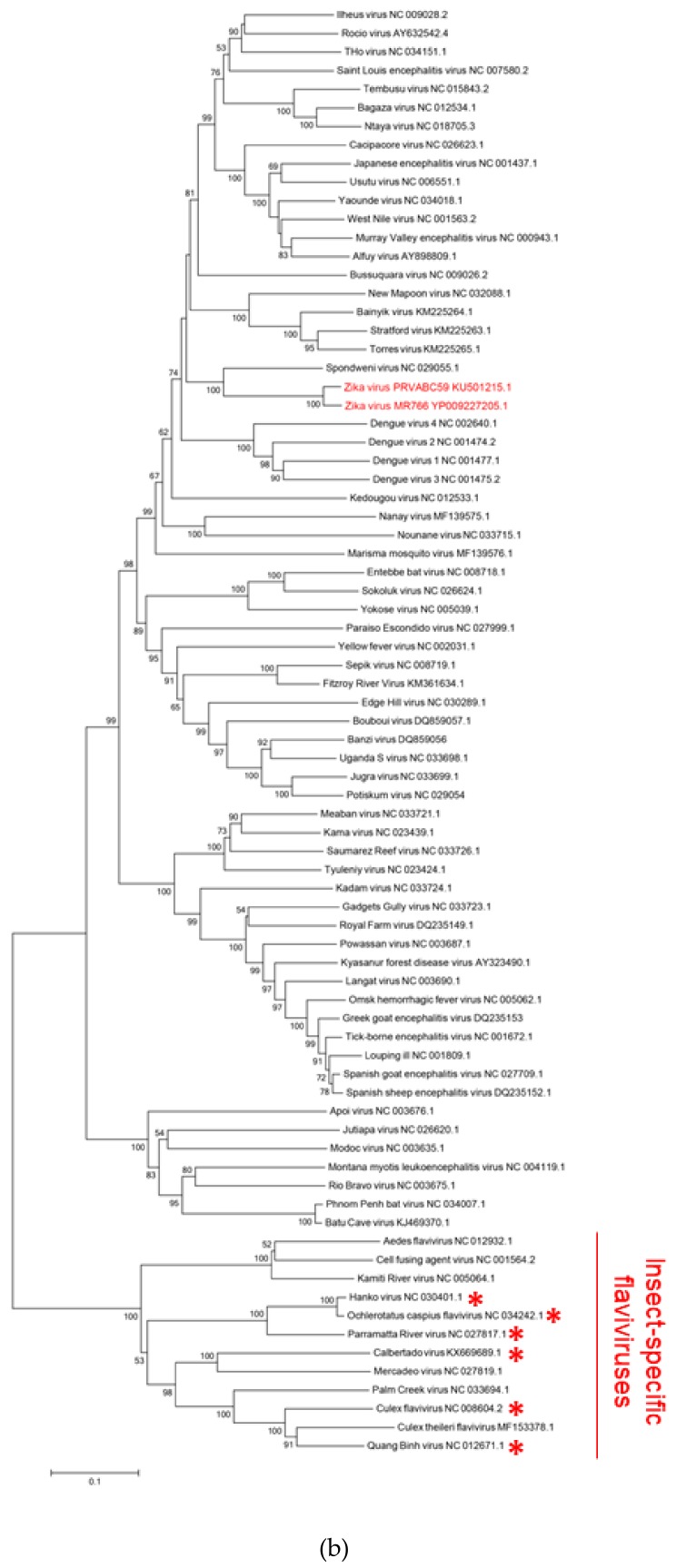

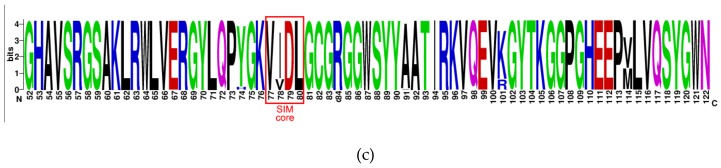
The putative SUMO-interacting motif (SIM) at the *N*-terminal domain of non-structural 5 (NS5) protein is highly conserved among flaviviruses and among pre-epidemic and epidemic Zika virus (ZIKV) strains. (**a**) Multiple sequence alignment of the NS5 amino acid sequences around the putative SIM of 78 representative flaviviruses with complete genome sequences available in GenBank (accessed on 1 May 2018) showing conserved amino acid sequences (VIDL or VVDL) of the putative SIM core among most (72/78, 92.3%) of the flaviviruses (labelled as “SIM core” in red). The amino acid positions (52 to 122) represent those of the epidemic ZIKV-PR (Puerto Rico strain PRVABC59, accession number KU501215). (**b**) Phylogenetic analysis of the NS5 protein of the 78 flaviviruses. The tree was constructed by the neighbor-joining method using MEGA 6.0 software, with bootstrap values being calculated from 500 trees. Bootstrap values lower than 50 were hidden. All flavivirus strains are labeled with their names followed by their accession numbers. In both (**a**) and (**b**), the epidemic ZIKV-PR and pre-epidemic ZIKV-MR766 (accession number YP_009227205) are highlighted in red. The 6 insect-specific flaviviruses which have different putative SIM core amino acids of CVDL, AVDL, or ALDL are marked by red asterisks. (**c**) The Sequence logo of the partial NS5 protein amino acid sequences (amino acid positions 52 to 122) of 414 ZIKV strains with complete genomes available in GenBank (accessed on 9 January 2018) show that the putative SIM at the *N*-terminal domain of the NS5 protein is highly conserved among pre-epidemic and epidemic ZIKV strains. The red box represents the putative SIM core at amino acid positions 77 to 80. The height of each stack is measured in “bits” of information, with each amino acid ordered from the most frequent to least frequent.

To determine whether the SIM at the *N*-terminal domain of the NS5 protein of DENV was also present at a similar position in other flaviviruses, we performed multiple sequence alignment of the partial NS5 sequences around the putative SIM of 78 representative flaviviruses with complete genome sequences available in GenBank (accessed on 1 May 2018). As shown in Figure 1a, most (72/78, 92.3%) of these flaviviruses had a putative SIM at a similar position near the N-terminus of the NS5 protein. The amino acid sequences of the putative hydrophobic cores of these 72 SIMs were VVDL (63/78, 80.8%) or VIDL (9/78, 11.5%) (amino acid positions 77-80 of ZIKV-PR). The 6 flaviviruses which did not have the same putative core amino acid sequences were Calbertado virus (KX669689.1), Culex flavivirus (NC_008604.2), Quang Binh virus (NC_012671.1), Hanko virus (NC_030401.1), Ochlerotatus caspius flavivirus (NC_034242.1), and Parramatta River virus (NC_027817.1). Instead of VVDL or VIDL, the putative core amino acid sequences of these 6 flaviviruses were CVDL (3/78, 3.8%), AVDL (2/78, 2.6%), or ALDL (1/78, 1.3%). Phylogenetic tree based on the full length NS5 amino acid sequences of these 78 viruses showed that these 6 viruses cluster together and represent insect-specific flaviviruses (Figure 1b). As the SUMO proteins of insects may be different from those of human and mammals, the motifs of these 6 flaviviruses might still be potential SIMs for insect SUMO proteins [20]. Overall, these genomic data showed that the putative SIM at the *N*-terminal domain of the NS5 protein is highly conserved among flaviviruses.

### 2.2. The Putative SIM at the N-Terminal Domain of the NS5 Protein is Conserved among Pre-Epidemic and Epidemic ZIKV Strains

ZIKV was considered to be relatively confined to Africa and some parts of Southeast Asia prior to the outbreak in Yap Island in 2007 [2]. We previously compared the genomes of pre-epidemic and epidemic ZIKV strains and found several amino acid mutations at the NS5 protein [1]. To investigate whether the putative SIM is conserved among pre-epidemic and epidemic ZIKV strains, we performed multiple sequence alignment of the NS5 sequences of all ZIKV strains (*n* = 414) with complete genomes available in GenBank (accessed on 9 January 2019). As shown in Figure 1c, the putative SIM at the *N*-terminal domain of the NS5 protein of all 414 ZIKV strains were conserved, with the core amino acid sequence being either VIDL (327/414, 79.0%) or VVDL (87/414, 21.0%). The sequence of the acidic residues flanking the hydrophobic core residues were also highly conserved among all 414 strains.

### 2.3. Molecular Docking Model of the Binding between the Putative ZIKV NS5 SIM and SUMO-1 Protein

**Figure 2:**
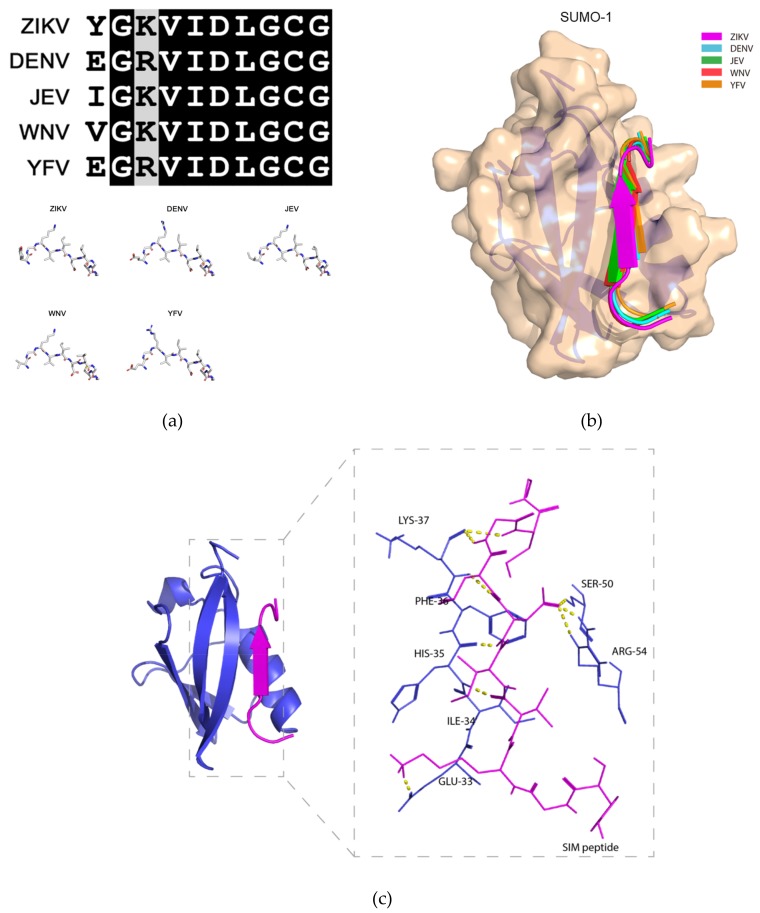
Molecular docking model of the binding between the putative non-structural 5 (NS5) SUMO-interacting motifs of flaviviruses and the SUMO-1 protein. (**a**) Top panel: multiple sequence alignment of the amino acid sequences of the putative NS5 protein SUMO-interacting motifs (SIM) of Zika virus (ZIKV), dengue virus (DENV) (serotype 3), Japanese encephalitis virus (JEV), West Nile virus (WNV), and yellow fever virus (YFV). Bottom panel: Stick representation of the structural similarities among the 5 flaviviruses’ putative NS5 SIM peptides. (**b**) Schematic representation of the binding between the SUMO-1 protein and the 5 flaviviruses’ putative NS5 SIM peptides. The SUMO-1 protein is shown in wheat and the NS5 SIM peptides are shown in different colors. (**c**) Ribbon representation showing the interacting amino acid residues of the putative ZIKV NS5 SIM peptide and the active sites of the SUMO-1 protein. The putative ZIKV NS5 SIM peptide and SUMO-1 protein are displayed in magenta and blue, respectively. The interacting residues are shown as sticks with hydrogen bonds represented by yellow dashed lines.

Next, we performed protein-peptide docking to obtain insights into the structural interactions between the SUMO protein and the putative NS5 SIMs of representative medically important flaviviruses, including ZIKV, DENV, JEV, WNV, and YFV. We used SUMO-1 protein in our molecular docking model as it is the isoform that has previously been shown to interact with flavivirus NS5 protein [19]. As shown in Figure 2a, the amino acid sequences of the putative NS5 SIMs of these flaviviruses are highly similar (Figure 2a, top panel). While there are a few differences among the *N*-terminal flanking amino acid residues, the overall structures of the putative SIMs remain highly similar (Figure 2a, bottom panel). The putative NS5 SIMs were all predicted to bind to the active site of the SUMO-1 protein located between the second beta-strand (β2) and the alpha helix, forming a parallel beta-sheet with β2 (Figure 2b). Using ZIKV as an example, our molecular docking model showed that multiple intermolecular hydrogen bonds were predicted to occur between the “VIDL” motif and the adjacent residues in the SUMO-1 protein (Figure 2c). These findings suggested a key role for the VIDL motif in the interaction between the putative NS5 SIMs of flaviviruses and the SUMO-1 protein.

### 2.4. The SUMO Inhibitor 2-D08 Significantly Inhibited the Replication of ZIKV and Other Medically Important Flaviviruses In Vitro

**Figure 3:**
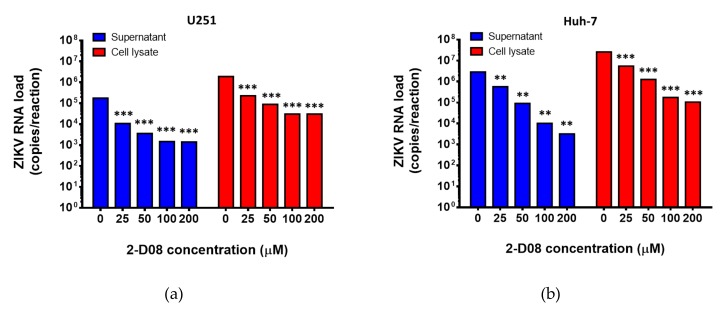
2-D08 inhibits the replication of Zika virus (ZIKV) in U251 and Huh-7 cells. Dose-dependent reduction of ZIKV RNA load was observed at 24 h after ZIKV infection (1.00 MOI) in (**a**) U251 and (**b**) Huh-7 cells with 0–200 μM of 2-D08. All experiments were performed in triplicate in three independent experiments for confirmation. ** denotes *P* < 0.01 and *** denotes *P* < 0.001 (compared with the DMSO control group by Student’s t-test). Data are presented as mean values ± standard deviations (error bars).

**Figure 4:**
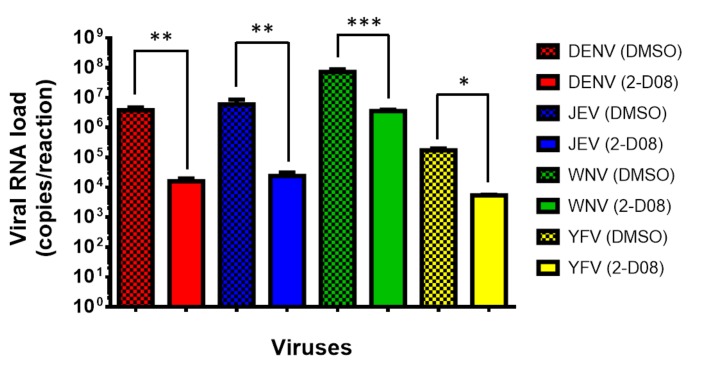
2-D08 inhibits the replication of medically important flaviviruses. Dose-dependent reduction of viral RNA load was observed at 48 h after virus infection (1.00 MOI) in U251 cells with 200 μM of 2-D08. All experiments were performed in triplicate in three independent experiments for confirmation. * denotes *P* < 0.05, ** denotes *P* < 0.01, and *** denotes *P* < 0.001 (compared with the DMSO control group by Student’s t-test). Data are presented as mean values ± standard deviations (error bars).

Based on these in silico results, we then investigated the biological relevance of SUMO modification of the ZIKV NS5 protein in virus replication by assessing the anti-ZIKV effects of a recently identified SUMO inhibitor (2-D08) in vitro [21]. The 50% cytotoxic concentration (CC_50_) of 2-D08 was determined to be >200 µM using the CellTiter-Glo® luminescent cell viability assay. Then, we treated ZIKV-infected U251 (human astrocytoma) cells with different doses of 2-D08. As shown in Figure 3a, 2-D08 significantly (*P* < 0.001) inhibited the replication of ZIKV (multiplicity of infection, MOI = 1.00) in both culture supernatant (~2.10 log10 copies/reaction at 200 µM) and cell lysate (~1.80 log10 copies/reaction at 200 µM) of U251 cells in a dose-dependent manner. The same dose-dependent anti-ZIKV effect was also observed in 2-D08-treated Huh-7 (human hepatoma) cells (*P* < 0.01) (culture supernatant: ~2.95 log10 copies/reaction at 200 µM; cell lysate: ~2.39 log10 copies/reaction at 200 µM) (Figure 3b). The half maximal inhibitory concentration (IC_50_) of 2-D08 in the viral load reduction assay ranged from 7.11-8.88 µM in U251 cells (selectivity index = >22.52 to >28.13) and 14.75-15.63 µM in Huh-7 cells (selectivity index = >12.80 to >13.56). Moreover, 2-D08 also significantly inhibited the replication of DENV, JEV, WNV, and YFV (Figure 4). These results corroborated our in silico prediction of the conserved putative SIM in these flaviviruses.

**Figure 5:**
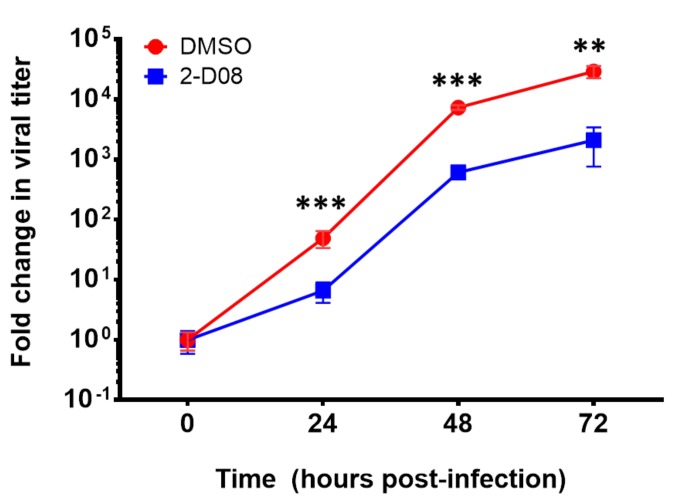
Differential viral kinetics of Zika virus (ZIKV) in U251 cells with or without 2-D08 from 0 to 72 hpost-infection. The ZIKV RNA load in the culture supernatant of the 2-D08-treated (100 µM) samples was consistently ~1.0 log10 lower than those of the DMSO-treated controls at different time points (24, 48, and 72 h post-infection). All experiments were performed in triplicate in three independent experiments for confirmation. ** denotes *P* < 0.01, and *** denotes *P* < 0.001 (compared with the DMSO control group at the same time point by Student’s *t*-test). Data are presented as mean values ± standard deviations (error bars).

**Figure 6:**
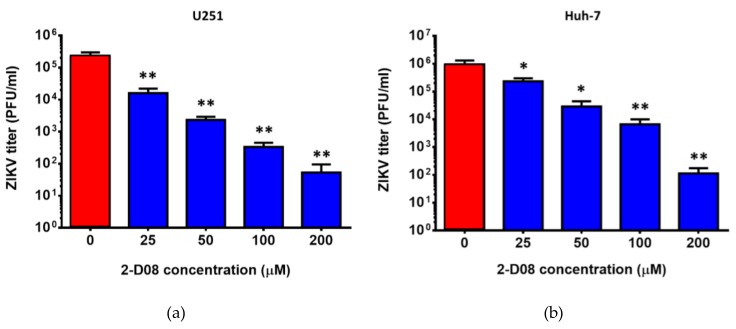
2-D08 inhibited infectious virus particles formation of ZIKV. Dose-dependent reduction of virus titer in the supernatant of ZIKV-infected (MOI = 1.00) (**a**) U251 and (**b**) Huh-7 cells with different concentrations of 2-D08 compared with DMSO (i.e., 0 μM of 2-D08) control. All experiments were performed in triplicate in three independent experiments for confirmation. * denotes *P* < 0.05 and ** denotes *P* < 0.01 (compared with the DMSO control group by Student’s t-test). Data are presented as mean values ± standard deviations (error bars).

**Figure 7:**
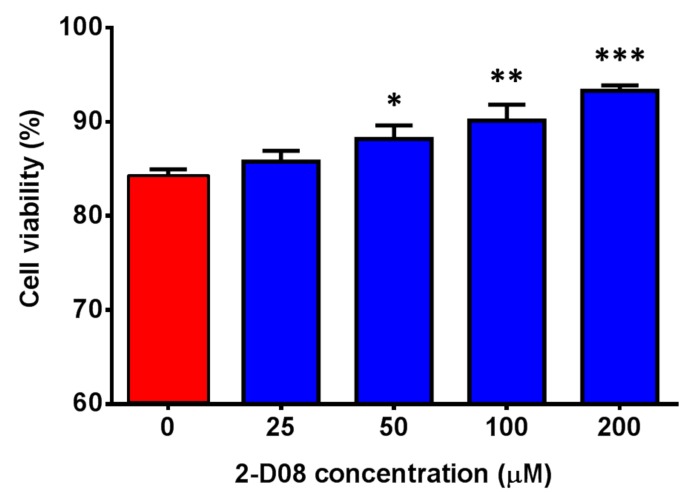
2-D08 provided cell protection effects against Zika virus (ZIKV) infection. Cell viability assay showing dose-dependent increase in the percentage of viable cells in 2-D08-treated U251 cells. All experiments were performed in triplicate in three independent experiments for confirmation. * denotes *P* < 0.05, ** denotes *P* < 0.01, and *** denotes *P* < 0.001 (compared with the DMSO control group by Student’s *t*-test). Data are presented as mean values ± standard deviations (error bars).

To evaluate the effect of 2-D08 on multi-cycle replication of ZIKV, we compared the differential viral kinetics of ZIKV in 2-D08-treated and DMSO-treated U251 cells at 24, 48, and 72 h post-infection. The fold change of 2-D08-treated U251 cells was consistently significantly lower (~10 times) than that of the DMSO-treated control cells over 72 h (*P* < 0.01) (Figure 5). In the plaque assay, 2-D08 significantly inhibited infectious virus particle formation in both U251 (IC_50_ = 10.16, selectivity index = >19.69) (*P* < 0.01) (Figure 6a) and Huh-7 cells (IC_50_ = 17.83, selectivity index = >11.22) (*P* < 0.05) (Figure 6b). In the cell protection assay, a dose-dependent increase in the percentage of viable cells was observed in 2-D08-treated U251 cells (*P* < 0.05) (Figure 7). Taken together, these results suggested that inhibition of SUMO modification reduces ZIKV replication in different cell types. The SUMO inhibitor 2-D08 effectively inhibited multi-cycle replication of ZIKV and provided cell protection effects against ZIKV infection in vitro.

### 2.5. SUMO Modification of the ZIKV NS5 Protein Is Required for NS5-Mediated Type I Interferon Signaling

**Figure 8:**
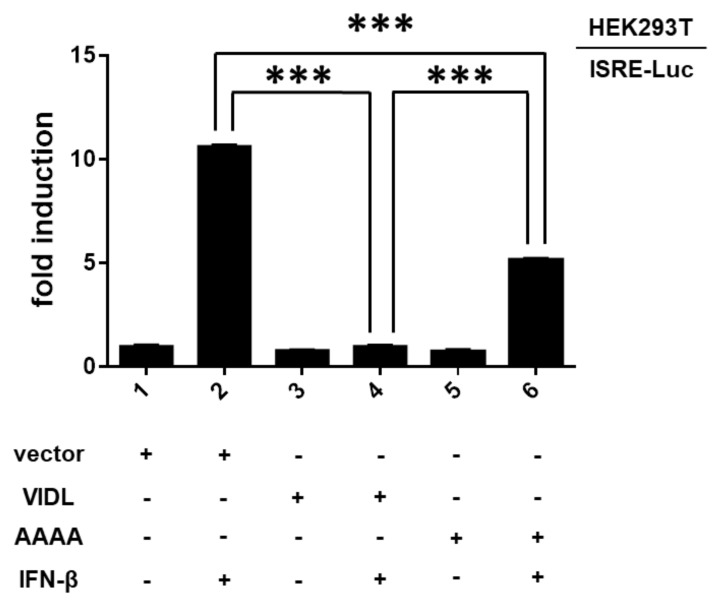
SUMO modification of the Zika virus (ZIKV) non-structural 5 (NS5) protein is required for NS5-mediated type I interferon signaling. Differential modulation of interferon-β signaling by wild type (VIDL) and SUMO-interacting motif (SIM)-mutated (AAAA) ZIKV NS5. HEK293T cells were transfected with an interferon-stimulated response element luciferase (ISRE-Luc) reporter plasmid and expression vectors of wild type (VIDL) or SIM-mutated ZIKV NS5 (AAAA). A Nano-Luc reporter vector was used as an internal control to normalize for transfection efficiency. At 6 hpost-transfection, 1000 U/mL interferon-β or phosphate-buffered saline control was added to the indicated wells. The cells were harvested 24 h after transfection and a dual-luciferase reporter assay using reagents supplied by Promega Corporation (Madison, WI, USA) was performed. All experiments were performed in triplicate in three independent experiments for confirmation. *** denotes *P* < 0.001. Data are presented as mean values ± standard deviations (error bars).

We have recently shown that the ZIKV NS5 protein can suppress type I interferon signaling by targeting signal transducer and activator of transcription 2 (STAT2) for proteasomal degradation [22]. STAT2 is known to form a heterodimer with STAT1 that interacts with interferon regulatory factor 9 to activate the interferon-stimulated response element (ISRE) in interferon-stimulated genes [23,24]. To investigate the effect of SUMO modification of the ZIKV NS5 protein on type I interferon signaling, we employed a luciferase reporter assay to compare the differential ISRE responses elicited by the wild type (VIDL) and a SIM-mutated (containing a “VIDL-to-AAAA” core amino acid substitution) ZIKV NS5. Consistent with our previous findings, interferon-β-induced activation of ISRE-dependent luciferase activity was significantly (*P* < 0.001) suppressed in HEK293T cells transfected with the wild-type ZIKV NS5 (Figure 8) [22]. In comparison, the suppression of interferon-β-induced activation of ISRE-dependent luciferase activity by the SIM-mutated ZIKV NS5 was significantly (*P* < 0.001) less than that of the wild type ZIKV NS5. These results suggested that SUMO modification of the ZIKV NS5 protein was essential for the NS5-mediated suppression of type I interferon signaling.

## 3. Discussion

Viruses utilize various types of post-translational modifications to evade host immune response and support their own replication. This may be achieved through host protein post-translational modification induced by the virus, or through post-translational modification of viral proteins. In the case of ZIKV, the ZIKV NS5 protein has been shown to selectively induce STAT2 ubiquitination and destabilization to suppress interferon signaling [22]. Recently, glutathionylation (glutathione covalently attached to a cysteine residue) of the ZIKV NS5 protein was reported to affect the protein’s guanylyltransferase and RNA-dependent RNA polymerase activities [25]. This study is the first to show that the ZIKV NS5 protein is a target for SUMO modification and that SUMO modification of the ZIKV NS5 protein leads to enhanced virus replication and suppressed type I interferon signaling.

SUMO proteins can modulate their target proteins’ functions via two mechanisms. First, SUMO proteins can covalently conjugate to their target proteins by binding to the lysine residues on the consensus sequence ψKxE (where ψ represents a large hydrophobic amino acid, K represents a lysine residue, x represents any amino acid, and E represents a glutamic acid residue) of the target proteins [11,26]. Second, the interacting surface of SUMO proteins can bind non-covalently with SIMs of the target proteins [27]. SIMs are usually a sequence of around 10 amino acids consisting of a hydrophobic core of V/I-x-V/I-V/I or V/I-V/I-x-V/I/L and a cluster of negatively charged residues flanking the core [27]. The DENV NS5 protein was recently identified to be a target for SUMO modification via binding between SUMO proteins with a SIM at the *N*-terminal domain of the NS5 protein [19]. This prompted us to investigate for the presence of a similar SIM in ZIKV and other flaviviruses, as the NS5 protein is highly conserved among flaviviruses [1].

Our in silico analysis showed that the putative NS5 SIM was indeed highly conserved among flaviviruses and among ZIKV. The majority of the 78 representative flaviviruses included in this analysis had a putative SIM at a similar position at the *N*-terminal domain of their NS5 protein as the DENV NS5 SIM, with the putative core amino acid residues of VVDL or VIDL. The 6 flaviviruses with putative core amino acid residues of CVDL, AVDL, or ALDL were insect-specific flaviviruses. Two SUMO paralogs, namely SUMO1 and SUMO3, have been predicted in insects [20]. In *Drosophila melanogaster*, SUMOylation is involved in various developmental processes, including embryogenesis, wing morphogenesis, neuronal development, immune response, and regulation of cell death [20]. Since the role of SUMO genes appears to be also evolutionarily conserved in insects, it would be interesting to investigate whether the putative core amino acid residues of these 6 insect-specific flaviviruses may also be targets for SUMO modification in insects. Notably, none of the amino acid mutations at the NS5 protein previously identified among pre-epidemic and epidemic ZIKV strains involved the SIM at the *N*-terminal domain [1]. Our molecular docking model predicted that the NS5 SIMs of medically important flaviviruses including ZIKV interacts with the SUMO-1 protein in a classical manner, in which the hydrophobic residues bind to the β2 strand of the SUMO-1 protein and the acidic residues flanking the hydrophobic core are responsible for strengthening the binding by interacting with the basic surface of the SUMO-1 protein [27].

The effects of SUMO modification of viral proteins on the replication of members of *Flaviviridae* are incompletely understood. A DENV replicon with a SUMOylation-defective SIM-mutated NS5 was found to be severely defective in virus replication [19]. Inhibition of SUMOylation of the NS5A protein of hepatitis C virus also resulted in reduced virus replication in vitro [28]. To investigate the biological relevance of SUMO modification of the ZIKV NS5 protein, we evaluated the effects exerted by the SUMO inhibitor 2-D08 on the replication of ZIKV and other medically important flaviviruses. We showed that inhibition of SUMO modification resulted in significantly reduced ZIKV replication in multiple cell types and in a dose-dependent manner. The SUMO inhibitor was effective in inhibiting multi-cycle replication of ZIKV. Moreover, using a luciferase reporter assay, we showed that SUMO modification of the ZIKV NS5 protein was essential for the protein to suppress the host’s type I interferon signaling. We have previously reported that ZIKV is highly susceptible to interferons in vitro and in vivo, and that the ZIKV NS5 protein can interact with and destabilize STAT2 to inhibit interferon signaling [22,29,30]. Similar to the DENV SIM-mutated NS5 which also failed to suppress the induction of STAT2-mediated host antiviral interferon signaling, the SIM-mutated ZIKV NS5 was also likely unstable and failed to efficiently suppress type I interferon signaling due to NS5 protein destabilization and degradation [19]. Importantly, 2-D08 also significantly reduced viral RNA load of DENV, JEV, WNV, and YFV in vitro, suggesting that SUMO inhibitors may be a broad-spectrum anti-flaviviral strategy.

The NS5 protein is the largest protein of ZIKV and has several enzyme activities that are important for virus replication [1,31]. The *N*-terminal part of the NS5 protein has guanylyltransferase, RNA guanine-N7-methyltransferase, and nucleoside 2’-*O*-methyltransferase activities, while the *C*-terminal part has RNA-dependent RNA polymerase activity [1,31]. The SIM identified in this study is located at the *N*-terminal methyltransferase domain of ZIKV and other flaviviruses. It would be interesting to investigate whether the enzyme activities of the NS5 protein would be affected by SUMO modification in future studies. Overall, the novel findings in this study may suggest SUMO modification of the viral NS5 protein to be an evolutionarily conserved and important post-translational modification process utilized by flaviviruses, which have a single open reading frame, to fine-tune their replicase protein expression and turnover in host cells in order to enhance virus replication and evade host antiviral response. The use of SUMO inhibitors as a form of host-targeting antiviral treatment for infections caused by ZIKV and other flaviviruses should also be evaluated in suitable animal models.

## 4. Materials and Methods

### 4.1. Genomic Characterization and Phylogenetic Analysis

The genome sequences of 414 ZIKV strains with reported NS5 sequences available in GenBank were included in this study (Supplementary Materials). These included strains collected from human, animals, and mosquitoes between 1947 and 2018. The complete NS5 sequences of other representative members of *Flaviviridae* according to the International Committee on Taxonomy of Viruses were also included for analysis in the study. Multiple sequence alignment of the amino acid sequences of the NS5 proteins of the flaviviruses was performed by ClustalX software version 1.83 and manually examined for any significant changes as we previously described [1]. The visualization of alignment results was performed using Weblogo [32,33]. Phylogenetic tree construction was performed using the neighbor-joining method using MEGA 6.0 software, with bootstrap values being calculated from 500 trees as we previously described [1].

### 4.2. ZIKV NS5 SIM and SUMO-1 Molecular Docking

The putative NS5 SIM 10-mer peptides were extracted from the NS5 protein crystal structures of ZIKV (PDB code: 5M5B), DENV (PDB code: 5JJS), JEV (PDB code: 4K6M), WNV (PDB code: 2OY0), and YFV (PDB code: 3EVA). The SUMO-1 crystal structure was separated from the SUMO-1-Daxx complex (PDB code: 4JWQ). These putative NS5 SIM peptides and the SUMO-1 protein structures were submitted to the HADDOCK2.2 webserver for protein-peptide docking simulation [34]. HADDOCK calculations were performed on the WeNMR GRID (http://www.wenmr.eu/) through the HADDOCK webserver Expert interface. The histidine protonation states were automatically determined by the WHATIF program which is embedded in the HADDOCK server on the WeNMR GRID. Residues of SUMO-1 implicated for binding were designated as active target [35]. Passive residues were defined automatically around the active residues by the HADDOCK webserver. The first stage of docking consisted of randomization of orientations and rigid body energy minimization, which yielded ~1000 solutions. The resulting structures were subjected to semi-rigid simulated annealing in torsion angle space and final refinement in Cartesian space with explicit solvent resulting in ~100-200 structures that were analyzed. The best scoring structure cluster was then selected for interaction analysis. Pymol was utilized to visualize the docked pose and interactions.

### 4.3. Virus Strains, Cell Lines, and Drug Compounds

ZIKV-PR (Puerto Rico strain PRVABC59, accession number KU501215) was isolated from a patient in the recent South American epidemic (kindly provided by Brandy Russell and Barbara Johnson, Centers for Disease Control and Prevention, USA). The virus strain was cultured and titrated as we previously described [6,36]. The U251, Huh-7, and HEK293T cell lines were obtained from American Type Culture Collection. The SUMO inhibitor 2-D08 was purchased from Merck Millipore (Burlington, MA, USA).

### 4.4. CellTiter-Glo® luminescent Cell Viability Assay

The CC_50_ of 2-D08 in U251 and Huh-7 cells and the cell protection effects of 2-D08 against ZIKV infection in these cell lines were determined by the CellTiter-Glo® luminescent cell viability assay (Promega Corporation) according to the manufacturer’s instructions and as previously described [36]. Briefly, for determination of CC_50_ of 2-D08 treatment without ZIKV infection, the cells were treated with different concentrations of 2-D08 (0–200 μM) for 48 h. For cell protection effects, the cells were infected by ZIKV-PR (MOI = 0.50) for 2 h and then treated with dimethyl sulfoxide (DMSO) (i.e., 0 μM of 2-D08) or up to 200 μM of 2-D08 for 48 h. After 48 h, the reconstituted CellTiter-Glo® reagent was added to the cells according to the manufacturer’s instructions. The luminescent signal was detected by the Victor X3 2030 Multilabel Reader (PerkinElmer, Inc., Waltham, MA, USA) according to the manufacturer’s instructions.

### 4.5. Viral Load Reduction Assay and Plaque Assay

The effect of 2-D08 against ZIKV was evaluated in cell culture as previously described with slight modifications [29,36,37,38]. Briefly, ZIKV-infected Huh-7 and U251 cells (MOI = 1.00) cells were treated with different concentrations of 2-D08 (0–200 μM) or DMSO (i.e., 0 µM of 2-D08) as control. The cell culture supernatants were then collected at 24 h post-infection, followed by total nucleic acid extraction and quantitative reverse transcription-PCR (qRT-PCR) or plaque assay as previously described [29,36,37,39]. Similarly, U251 cells infected with DENV, JEV, WNV, or YFV were treated with 2-D08 (200 μM) or DMSO and the cell culture supernatants were collected at 48 h post-infection for viral load reduction assay using previously described qRT-PCR assays [40,41,42]. For comparison of the differential viral kinetics of ZIKV in 2-D08-treated versus DMSO-treated U251 cells over time, ZIKV-infected (MOI = 0.10) U251 cells were treated with 100.0 µM of 2-D08 or DMSO (control). The cell culture supernatants were collected at 0, 24, 48, and 72 h post-infection, followed by total nucleic acid extraction and qRT-PCR.

### 4.6. Luciferase Reporter Assay

HEK293T (human embryonic kidney) cells were transfected with an ISRE luciferase (ISRE-Luc) reporter plasmid and expression vectors of ZIKV NS5 proteins as previously described [22]. A Nano-Luc reporter vector was used as an internal control to normalize for transfection efficiency. At 6 h post-transfection, 1000 U/ml interferon-β was added to the indicated wells. The cells were harvested 24 h after transfection and a dual-luciferase reporter assay using reagents supplied by Promega Corporation was performed as we previously described [22,43,44]. The data were recorded with the Victor X3 2030 Multilabel Reader (PerkinElmer) according to the manufacturer’s instructions. *N*-terminal flag-tagged NS5 genes from ZIKV were individually cloned into pCAGEN vector between XhoI and NotI restriction sites as we previously described [22]. The mutation of NS5 plasmid was done by GenScript (Piscataway, NJ, USA).

### 4.7. Statistical Analysis

All data were analyzed with GraphPad Prism software (GraphPad Software, Inc, San Diego, CA, USA). Statistical comparisons between different groups were performed by the Student’s *t*-test as we described previously [29,30,45]. *P*-value of <0.05 was considered as statistically significant. Selectivity index was defined as CC_50_/IC_50_.

## 5. Conclusions

A highly conserved putative NS5 SIM was found in ZIKV and most other flaviviruses. The SUMO inhibitor 2-D08 significantly reduced the replication of ZIKV and other medically important flaviviruses. These findings enhanced our understanding in the post-translational modifications utilized by flaviviruses in their replication cycles and provided novel insights into developing potential host-targeting treatment strategies for treating flavivirus infections.

## Supplementary Materials

Supplementary Materials can be found at http://www.mdpi.com/1422-0067/20/2/392/s1.

## Abbreviations

HEK293T	Human embryonic kidney epithelial cell line
Huh-7	Human hepatoma cell line
ISRE	Interferon-stimulated response element
MOI	Multiplicity of infection
SIM	SUMO-interacting motif
STAT2	Signal transducer and activator of transcription 2
SUMO	Small ubiquitin-like modifier
U251	Human astrocytoma cell line
ZIKV	Zika virus
